# Composition, Clinical Efficiency, and Mechanism of NHC-Approved “Three Chinese Medicines and Three Chinese Recipes” for COVID-19 Treatment

**DOI:** 10.3389/fphar.2021.781090

**Published:** 2022-02-04

**Authors:** Ke-Yao Xia, Zeyuan Zhao, Taif Shah, Jing-Yi Wang, Zulqarnain Baloch

**Affiliations:** ^1^ Faculty of Traditional Chinese Medicine, Macau University of Science and Technology, Taipa, Macao SAR, China; ^2^ Faculty of Life Science and Technology, Yunnan Provincial Center for Molecular Medicine, Kunming University of Science and Technology, Kunming, China

**Keywords:** coronaviruses, COVID-19, traditional Chinese medicine (TCM), san-yao san-fang, three Chinese medicine and three Chinese recipes

## Abstract

Traditional Chinese medicines (TCMs) have been regularly prescribed to treat and prevent diseases for thousands of years in the eastern part of the Asian continent. Thus, when the coronavirus disease 2019 (COVID-19) epidemic started, TCM was officially incorporated as a strategy by the National Health Commission (NHC) for the treatment of COVID-19 infection. TCMs were used to treat COVID-19 and had a significant effect on alleviating symptoms, delaying disease progression, improving the cure rate, and reducing the mortality rate in China. Therefore, China’s National Health Commission officially approved Qingfei Paidu decoction, Xuanfei Baidu decoction, Huashi Baidu decoction, Lianhua Qingwen capsules, Jinhua Qinggan granules, and Xuebijing for COVID-19 treatment. This review evaluates and summarizes the use of TCMs against infectious diseases and the composition, clinical efficacy, and mechanisms of the NHC-approved “three Chinese medicines and three Chinese recipes” for COVID-19 treatment. The three Chinese medicines and three Chinese recipes have been demonstrated to be highly effective against COVID-19, but there is a lack of *in vivo* or *in vitro* evidence. Most of the available data related to the potential mechanism of the three Chinese medicines and three Chinese recipes is based on virtual simulation or prediction, which is acquired *via* molecular docking and network pharmacology analysis. These predictions have not yet been proven. Therefore, there is a need for high-quality *in vivo* and *in vitro* and clinical studies by employing new strategies and technologies such as genomics, metabolomics, and proteomics to verify the predicted mechanisms of these drug’s effects on COVID-19.

## Introduction

Coronavirus disease 2019 (COVID-19) is caused by the severe acute respiratory syndrome coronavirus 2 (SARS-CoV-2), which was first reported in December 2019 in Wuhan, China. As this disease spread rapidly due to its strong contagiousness, long incubation periods, and high pathogenicity, the World Health Organization (WHO) first declared the epidemic as a public health emergency of international concern on January 30, 2020. Moreover, COVID-19 was declared a pandemic on March 11, 2020. The numbers of confirmed cases and deaths have already reached 215,882,875 and 4,492,818, respectively, on August 29, 2021, worldwide.

Generally, the control measures against the COVID-19 pandemic can be divided into three aspects: minimizing the risk of transmission, improving prevention, and treatment. Keeping a social distance, wearing masks, taking vaccines, etc. can minimize the spread of COVID-19. Currently, the main western drugs used in the COVID-19 treatment are remdesivir, favipiravir, chloroquine, etc. (Maclachlan et al., XXX); their effectiveness is question mark. Unfortunately, there are no specific drugs available for the treatment of COVID-19 (Huang et al., 2020a). Although many vaccines are available, their effectiveness decreases as the virus mutates, such as the SARS-CoV-2 variant Delta (Planas et al., 2021). So, there is an urgent need for new therapeutics that can effectively treat COVID-19 and are unaffected by the threat of viral mutation.

Coronaviruses (CoVs) are a large group of viruses (Fehr and Perlman, 2015), consisting of hundreds of viruses, that generally causes mild to moderate respiratory system infections, such as flu, common cold, etc. Traditional Chinese medicine (TCM) has successfully treated infectious and non-infectious diseases for thousands of years. During the COVID-19 outbreak in Wuhan, China’s national government appointed 3,100 TCM doctors at Wuhan and appointed TCM experts to screen and formulate effective TCM prescriptions for COVID-19 (Ren et al., 2020a). TCM had a significant effect on COVID-19, alleviating symptoms, delaying disease progression, improving cure rate, and reducing mortality rate (Ren et al., 2020b). Therefore, TCM has attained significant practical experience and a basis for long-term epidemic or pandemic prevention and treatment. According to the latest guidelines on the diagnosis and treatment of novel coronavirus pneumonia (interim 8th version) published by the China National Health Commission (NHC), “three Chinese medicines and three Chinese recipes” (三药三方) have been officially approved for COVID-19 treatment, including Qingfei Paidu decoction (QFPD), Xuanfei Baidu decoction (XFBD), Huashi Baidu decoction (HSBD), Lianhua Qingwen capsules (LHQW), Jinhua Qinggan granules (JHQG), and Xuebijing injection (XBJ) (Commission, 2019). This review evaluates and summarizes the use of TCMs against infectious diseases and the composition, clinical efficacy, and mechanisms of NHC-approved three Chinese medicines and three Chinese recipes for COVID-19 treatment.

## TCM Application Against Infectious Diseases

Based on the valuable experience of understanding life, maintaining health, production, and medical practice, TCM has been regularly prescribed to treat and prevent diseases for thousands of years in the eastern part of the Asian continent (Liu et al., 2020a). According to literature, TCM prescription against infectious diseases was first described in Huangdi Neijing in 200 BC (Cavalieri and Rotoli, 1997). The classical TCM book, Shanghan Lun (Treatise on Cold Damage Diseases as the translation), described a system of identifying diseases by six syndromes, with relevant symptoms and pulses for the diagnosis and appropriate TCM prescriptions for curing infectious diseases (Chen et al., 2009). The first TCM application against infectious diseases (febrile diseases) was reported by Zhongjing Zhang (150–219 AD) during the Han dynasty (202 BC–220 AD) (Chen et al., 2009). He had prescribed different formulas to treat febrile diseases, i.e., Mahuang decoction, prescribed against cough, myalgia, and chills, and Maxingshigan decoction, prescribed against high fever and dyspnea. Interestingly, clinical applications of his thousands of years of formulas are still popular in China (Hsieh et al., 2012).

Recently, artemisinin, an active ingredient extracted from the plant Artemisia carvifolia that is effective against malaria, was discovered in an ancient TCM book, Zhou Hou Bei Ji Fang (the Eastern Jin Dynasty 317–420 AD) by Tu Youyou, who won the Nobel Prize in Physiology and Medicine in 2015 (Wright et al., 2010). Another TCM book, Wen Bing Tiao Bian, written by Wu Jutong (1758–1836 AD), proposed for the first time that Yinqiao powder, Xuanbai Chengqi decoction, and Sanren decoction can be used for the treatment of infectious diseases (Huang, 2002). TCM, such as Lianhua Qingwen extracts, has been used to treat influenza viruses (Ding and Giannoumis, 2017). During the 2002–2003 severe acute respiratory syndrome (SARS) outbreaks in China, TCM was widely used in combination with Western medicine to treat SARS cases. According to the reports, the combination of TCM and Western medicine can effectively improve clinical symptoms and hypoxemia and protect the functions of the lung and heart (Jia and Gao, 2003). In short, TCM has been shown to possess antiviral activities against various viruses including herpes simplex virus, hepatitis B and C viruses, influenza virus, human immunodeficiency virus, SARS-CoV, MERS-CoV, etc. (Huang et al., 2021).

## Three Chinese Medicines and Three Chinese Recipes

Based on the successful application of TCMs against SARS and H1N1 influenza, China NHC has suggested TCM administration as a treatment strategy against COVID-19 (Wang and Qi, 2020). However, TCM has been officially incorporated as a strategy by the NHC for the diagnosis and treatment of COVID-19. The Chinese COVID-19 treatment method was also accepted by the director-general of the WHO senior adviser, Bruce Elwald (Zhao et al., 2021a). When we searched with the keywords “Chinese medicine” and “COVID-19” on September 12, 2021, 4,174 articles had been published on PubMed, which shows that there has been a lot of research conducted based on TCMs and reveals that TCM has recovered COVID-19 patients’ symptoms and stopped the progression of the disease from mild to severe (Zhou et al., 2021a).

Three Chinese medicines and three Chinese recipes were indeed used from the onset of COVID-19 in different parts of China, but JHQG, LHQW, and XBJ were first proposed in the national guidelines for the diagnosis and treatment of novel coronavirus pneumonia of COVID-19 (interim 4th version) on January 27, 2020 (National Health Commission of the People’s Republic of China, 2020a). However, QFPD, XFBD, and HSBD were first mentioned in the national guidelines for the diagnosis and treatment of novel coronavirus pneumonia of COVID-19 (interim 6th version) on February 28, 2020 (National Health Commission of the People’s Republic of China, 2020b). Then, on March 18, 202, these six TCMs were named as the “three Chinese medicines and three Chinese recipes” (Medicineo NAoTC XXXd). These medicines’ applications were classified according to disease severity.

QFPD was developed by Ge YW, based on four prescriptions (MXSG, SGMH, XCH, and WLS) from the classic TCM book by Shang Han Lun, a distinguished researcher at the China Academy of Chinese Medical Sciences (Medicineo NAoTC XXXb). A total of 214 COVID-19 patients received QFPD in four provinces, with an effectiveness rate of more than 90% (Medicineo NAoTC XXXc). Thus, the National Administration of TCM recommended QFPD for COVID-19 treatment on February 7, 2020 (Medicineo NAoTC XXXe). Furthermore, guidelines on the diagnosis and treatment of novel coronavirus disease (interim 6th version) published by the NHC recommend QFPD for the treatment of COVID-19 (National Health Commission of the People’s Republic of China, 2020b). XBFD was designed based on MXSG, MXYG, QJWJ, and TLDZXF prescriptions from the classic TCM book named Shang Han Lun. Chinese academician Zhang Zhongli and professor Liu and his team in Wuhan first proposed XFBD treatment for moderate and severe COVID-19 patients. A study was conducted in Traditional Chinese Medicine Wuhan Hospital in Hubei Province. The results showed that the Xuanfei-Baidu formula had a significant anti-inflammatory effect apart from improving the lymphocyte count of COVID-19 patients (Medicineo NAoTC XXXe). Therefore, XFBD was recommended by NHC in the guidelines on the diagnosis and treatment of novel coronavirus pneumonia to treat COVID-19 on February 28, 2020 (National Health Commission of the People’s Republic of China, 2020b). HSBD was developed by Chinese academician Huang and his team based on MXSG, HXZQ, XBCQ, and TLDZXF ancient TCM prescriptions (Medicineo NAoTC XXXa). HSBD was administered to 75 severe COVID-19 patients, and it was found that HSBD significantly recovered COVID-19 patients, and their nucleic acid turned negative (Medicine NAoTC Huang, Luqi). HSBD was first recommended in the guidelines (interim 6th version) (National Health Commission of the People’s Republic of China, 2020b). LHQW is one of the TCMs, available in granular and capsule form, developed by Shijiazhuang Yiling Pharmaceutical, which has been commonly used against different viruses due to its antiviral and immunomodulatory effects. However, it became famous in 2003 during the SARS outbreak when it played a significant role in combatting SARS during 2002–2003 in China (Jia et al., 2015). Furthermore, LHQW was recommended by the NHC for influenza and other respiratory infections in 2004 (Jia et al., 2015; Zhong et al., 2016). It was also recommended for the treatment of COVID-19 in the guidelines (interim 4th version) issued by the NHC on January 27, 2020 (National Health Commission of the People’s Republic of China, 2020a). JHQG has been derived from Maxing Shigan decoction written in Shang Han Lun, a classic ancient TCM book written by Zhang Zhongjing, and Yinqiao powder in Wen Bing Tiao Bian by WU Jutong (Lin et al., 2020). Jinhua Qinggan granules is a Chinese medicine prescription developed by experts working in the Beijing Administration of Traditional Chinese Medicine. JHQG was recommended to use against an avian influenza A (H1N1) virus and was very effective against H1N1 (China MoHotPsRo, 2009). Additionally, JHQG could also improve the immune function of influenza patients (Qi and Wang, 2016). Therefore, NHC also recommended JHQG for the treatment of COVID-19 in the guidelines (interim 4th version) issued by the NHC on January 27, 2020 (National Health Commission of the People’s Republic of China, 2020a). XBJ injection is a patent TCM developed in 2003 and is mainly based on a classic TCM prescription called Xuefu Zhuyu decoction. XBJ was first used to treat influenza, MERS, dengue, and Ebola (Tong et al., 2020). XBJ has also been recommended to treat critical COVID-19 cases in the guidelines (interim 4th version) (National Health Commission of the People’s Republic of China, 2020a).

## The Composition of Three Chinese Medicines and Three Chinese Recipes

We compared the composition of the six most effective recipes of three Chinese medicines and three Chinese recipes for COVID-19 treatment (Table 1). There are 50 total TCM compounds; among them, 47 are Chinese herbs, two fungi, and one mineral; of these, 14 components are common in five NHC-approved drugs for COVID-19. Ephedrae Herba, Glycyrrhizae Radix et Rhizoma (甘草), Armeniacae Semen Amarum (苦杏仁), and Gypsum Fibrosum (石膏) are the common among HSBD, LHQW, XFBD, LHQW, and JHQG. Lonicerae Japonicae Flos (金银花), Forsythiae Fructus (连翘), and Menthae Haplocaltcis Herba (薄荷) are common herbs of LHQW and JHQG. Atractylodis Rhizoma (苍术) and Descurainiae Semen Lepidii Semen (葶苈子) are common in HSBD and XFBD. Scutellariae Radix (黄芩) is the common herb in QFPD and JHQG. Ageratum rugose (藿香) is common in HSBD and QFPD. Atractylodis Rhizoma (苍术) is common in HSBD and XFBD. Pogostemonis Herba (广藿香) is common in XFBD and LHQW. Artemisiae Annuae Herba (青蒿) is common in XFBD and JHQG. Descurainiae Semen Lepidii Semen (葶苈子) is common in HSBD and XFBD. Poria (茯苓) is common in HSBD and QFPD. The other 37 components are added individually in different drugs. We observed that the compositions of oral drugs are complex compared to injectables. Interestingly, the composition of injectables is different compared to other drugs (Table 1).

**TABLE 1:** Composition of the National Health Commission (NHC)-approved Chinese drugs against COVID-19.

Ingredients (Chinese)*	HSBD	QFPD	XFBD	LHQW	JHQG	XBJ	References
Ephedrae Herba (麻黄)	√	√	√	√	√	×	Tao et al. (2013), Ding et al. (2017), Liu et al. (2020b), National Health Commission of the People’s Republic of China (2020c), Pan et al. (2020), Zhuang et al. (2020)
Glycyrrhizae Radix et Rhizoma (甘草)	√	√	√	√	√	×	Tao et al. (2013), Ding et al. (2017), Liu et al. (2020b), National Health Commission of the People’s Republic of China (2020c), Pan et al. (2020), Zhuang et al. (2020)
Armeniacae Semen Amarum (苦杏仁)	√	√	√	√	√	×	Tao et al. (2013), Ding et al. (2017), Liu et al. (2020b), National Health Commission of the People’s Republic of China (2020c), Pan et al. (2020), Zhuang et al. (2020)
Cinnamomi Ramulus (桂枝)	×	√	×	×	×	×	National Health Commission of the People’s Republic of China (2020c), Pan et al. (2020)
Alismatis Rhizoma (泽泻)	×	√	×	×	×	×	National Health Commission of the People’s Republic of China (2020c), Pan et al. (2020)
Atractylodis Macrocephalae Rhizoma (白术)	×	√	×	×	×	×	National Health Commission of the People’s Republic of China (2020c), Pan et al. (2020)
Bupleuri Radix (柴胡)	×	√	×	×	×	×	National Health Commission of the People’s Republic of China (2020c), Pan et al. (2020)
Scutellariae Radix (黄芩)	×	√	×	×	√	×	Liu et al. (2020b), National Health Commission of the People’s Republic of China (2020c), Pan et al. (2020), Zhuang et al. (2020)
Pinelliae Rhizoma Praeparatum Cum Zingibere et Alumine (姜半夏)	×	√	×	×	×	×	National Health Commission of the People’s Republic of China (2020c), Pan et al. (2020)
Zingiberis Rhizoma Recens(生姜)	×	√	×	×	×	×	National Health Commission of the People’s Republic of China (2020c), Pan et al. (2020)
Asteris Radix et Rhizoma (紫菀)	×	√	×	×	×	×	Commission (2020c), Pan et al. (2020)
Farfarae Flos (款冬花)	×	√	×	×	×	×	National Health Commission of the People’s Republic of China (2020c), Pan et al. (2020)
Belamcandae Rhizoma (射干)	×	√	×	×	×	×	National Health Commission of the People’s Republic of China (2020c), Pan et al. (2020)
Asari Radix et Rhizoma (细辛)	×	√	×	×	×	×	National Health Commission of the People’s Republic of China (2020c), Pan et al. (2020)
Dioscoreae Rhizoma (山药)	×	√	×	×	×	×	National Health Commission of the People’s Republic of China (2020c), Pan et al. (2020)
Aurantii Fructus Immaturus (枳实)	×	√	×	×	×	×	National Health Commission of the People’s Republic of China (2020c), Pan et al. (2020)
Citri Reticulatae Pericarpium (陈皮)	×	√	×	×	×	×	National Health Commission of the People’s Republic of China (2020c), Pan et al. (2020)
Ageratum rugose (藿香)	√	√	×	×	×	×	National Health Commission of the People’s Republic of China (2020c), Pan et al. (2020)
Coicis Semen (薏苡仁)	×	×	√	×	×	×	National Health Commission of the People’s Republic of China (2020c), Pan et al. (2020)
Atractylodis Rhizoma (苍术)	√	×	√	×	×	×	National Health Commission of the People’s Republic of China (2020c), Pan et al. (2020)
Pogostemonis Herba (广藿香)	×	×	√	√	×	×	Tao et al. (2013), Ding et al. (2017), Commission (2020c), Pan et al. (2020), Zhuang et al. (2020)
Artemisiae Annuae Herba (青蒿)	×	×	√	×	√	×	Liu et al. (2020b), National Health Commission of the People’s Republic of China (2020c), Pan et al. (2020), Zhuang et al. (2020)
Polygoni Cuspidati Rhizoma et Radix (虎杖)	×	×	√	×	×	×	National Health Commission of the People’s Republic of China (2020c), Pan et al. (2020)
Verbenae Herba (马鞭草)	×	×	√	×	×	×	National Health Commission of the People’s Republic of China (2020c), Pan et al. (2020)
Phragmitis Rhizoma (芦根)	×	×	√	×	×	×	National Health Commission of the People’s Republic of China (2020c), Pan et al. (2020)
Descurainiae Semen Lepidii Semen (葶苈子)	√	×	√	×	×	×	National Health Commission of the People’s Republic of China (2020c), Pan et al. (2020)
Citri Grandis Exocarpium (化橘红)	×	×	√	×	×	×	National Health Commission of the People’s Republic of China (2020c), Pan et al. (2020)
Mangnoliae Officinalis Cortex (厚朴)	√	×	×	×	×	×	National Health Commission of the People’s Republic of China (2020c), Pan et al. (2020)
Tsaoko Fructus (草果)	√	×	×	×	×	×	National Health Commission of the People’s Republic of China (2020c), Pan et al. (2020)
Pinelliae Rhizoma Praeparatum (法半夏)	√	×	×	×	×	×	National Health Commission of the People’s Republic of China (2020c), Pan et al. (2020)
Rhei Radix et Rhizoma (大黄)	√	×	×	√	×	×	Tao et al. (2013), Ding et al. (2017), Commission (2020c), Pan et al. (2020), Zhuang et al. (2020)
Astragali Radix (黄芪)	√	×	×	×	×	×	National Health Commission of the People’s Republic of China (2020c), Pan et al. (2020)
Paeoniae Radix Rubra (赤芍)	√	×	×	×	×	√	Zuo et al. (2017), Zuo et al. (2019), National Health Commission of the People’s Republic of China (2020c), Pan et al. (2020), Zhuang et al. (2020)
Lonicerae Japonicae Flos (金银花)	×	×	×	√	√	×	Tao et al. (2013), Ding et al. (2017), Liu et al. (2020b), Pan et al. (2020), Zhuang et al. (2020)
Forsythiae Fructus (连翘)	×	×	×	√	√	×	Tao et al. (2013), Ding et al. (2017), Liu et al. (2020b), Pan et al. (2020), Zhuang et al. (2020)
Fritillariae Thunbergii Bulbus (浙贝母)	×	×	×	×	√	×	Liu et al. (2020b), Pan et al. (2020), Zhuang et al. (2020)
Anemarrhenae Rhizoma (知母)	×	×	×	×	√	×	Liu et al. (2020b), Pan et al. (2020), Zhuang et al. (2020)
Arctii Fructus (牛蒡子)	×	×	×	×	√	×	Liu et al. (2020b), Pan et al. (2020), Zhuang et al. (2020)
Menthae Haplocaltcis Herba (薄荷)	×	×	×	√	√	×	Tao et al. (2013), Ding et al. (2017), Liu et al. (2020b), Pan et al. (2020), Zhuang et al. (2020)
Isatidis Radix (板蓝根)	×	×	×	√	×	×	Tao et al. (2013), Ding et al. (2017), Pan et al. (2020), Zhuang et al. (2020)
Dryopteridis Crassirhizomatis Rhizoma (绵马贯众)	×	×	×	√	×	×	Tao et al. (2013), Ding et al. (2017), Pan et al. (2020), Zhuang et al. (2020)
Houttuyniae Herba (鱼腥草)	×	×	×	√	×	×	Tao et al. (2013), Ding et al. (2017), Pan et al. (2020)
Rhodiolae Crenulatae Radix et Rhizoma (红景天)	×	×	×	√	×	×	Tao et al. (2013), Ding et al. (2017), Pan et al. (2020)
Carthami Flos (红花)	×	×	×	×	×	√	Zuo et al. (2017), Zuo et al. (2019), Pan et al. (2020), Zhuang et al. (2020)
Chuanxiong Rhizoma (川芎)	×	×	×	×	×	√	Zuo et al. (2017), Zuo et al. (2019), Pan et al. (2020), Zhuang et al. (2020)
Salviae Miltiorrhizae Radix et Rhizoma (丹参)	×	×	×	×	×	√	Zuo et al. (2017), Zuo et al. (2019), Pan et al. (2020), Zhuang et al. (2020)
Angelicae Sinensis Radix (当归)	×	×	×	×	×	√	Zuo et al. (2017), Zuo et al. (2019), Pan et al. (2020), Zhuang et al. (2020)
Gypsum Fibrosum (石膏)	√	√	√	√	√	×	Tao et al. (2013), Ding et al. (2017), Liu et al. (2020b), National Health Commission of the People’s Republic of China (2020c), Pan et al. (2020), Zhuang et al. (2020)
Poria (茯苓)	√	√	×	×	×	×	National Health Commission of the People’s Republic of China (2020c), Pan et al. (2020)
Polyporus (猪苓)	×	√	×	×	×	×	National Health Commission of the People’s Republic of China (2020c), Pan et al. (2020)

Of these herbs, Armeniacae Semen Amarum and Ephedrae Herba are two of the most commonly used herbs for the treatment of mild, moderate, and severe COVID-19 infection (Ang et al., 2020; Huang et al., 2021). In addition, Scutellariae Radix, Forsythiae Fructus, Ageratum rugose, and Atractylodis Rhizoma are used to cure mild and moderate COVID-19 infection (Ang et al., 2020; Huang et al., 2021). Notably, Glycyrrhizae Radix et Rhizoma is commonly used to cure mild, moderate, and severe COVID-19 patients (Ang et al., 2020; Huang et al., 2021). It has been reported that Lonicerae Japonicae Flos, Forsythiae Fructus, Verbenae Herba, and Carthami Flos have different types of active compounds that have therapeutic effects on COVID-19 (Huang et al., 2021; Niu et al., 2021).

## The Clinical Efficiency of Three Chinese Medicines and Three Chinese Recipes

The COVID-19 pandemic is still ongoing and affects individuals in different ways. Therefore, disease progression has been classified into three stages according to signs, symptoms, and disease severity, e.g., stage I, stage II, and stage III. Stage I begins with upper respiratory tract symptoms such as dry cough, fever, tiredness, etc. COVID-19 stage II is also called the pulmonary phase in which infected patients are diagnosed with pneumonia without hypoxia or with hypoxia. Normally, patients diagnosed with pneumonia with hypoxia need hospitalization and oxygen supplementation. Stage III, also known as the hyper-inflammation phase, in which an infected patient’s health suddenly worsens, typically develops acute respiratory distress syndrome (ARDS). Most of the COVID-19-related deaths occur in this phase. Keeping in view the importance of the COVID-19 infection developmental stages, the NHC has also issued recommendations for clinicians to use the three Chinese medicines and three Chinese recipes under specific signs and symptoms (Table 2). LHQW and JHQG have been recommended for use in patients with fatigue, fever, etc. XFBD and HSBD are recommended for treating moderate and severe cases, respectively. XBJ can be used only in critical cases, while QFPD is recommended for both non-critical and critical patients. Details of clinical applications of individual drugs are given below.

**TABLE 2:** Application of TCM against different symptoms of COVID-19 recommended by China National Health Commission (NHC) (National Health Commission of the People’s Republic of China, 2020c).

Symptoms	HSBD	QFPD	XFBD	LHQW	JHQG	XBJ
Cough	√	√	√	×	×	√
Fever	√	√	√	√	√	√
Short breath	√	√	√	×	×	√
Fatigue	√	√	×	√	√	√
Muscle or body aches	×	√	×	×	×	×
Headache	×	√	×	×	×	×
Sore throat	×	√	×	×	×	×
Nausea	√	√	√	×	×	×
Expectoration	√	√	√	×	×	√
Dyspnea	×	√	×	×	×	√
Shock	×	√	×	×	×	√

QFPD was used in 214 COVID-19 patients in four provinces and was found to have a therapeutic response rate of over 90% after comprehensive evaluation (Medicineo NAoTC XXXc). Different direct or indirect evidence-based studies have endorsed QFPD for the treatment of COVID-19 (Cao et al., 2020; Liang et al., 2020; Zhang et al., 2020a). Additionally, QFPD is the only TCM drug recommended for the treatment of all stages of COVID-19 cases. Thousands of COVID-19 patients have been recovered with QFPD administration, and it also stops the progression of the mild disease to the critical stage (Zhao et al., 2020; Zhong et al., 2020). In a retrospective multicenter study, 782 patients with mild-to-moderate COVID-19 were allocated to take QFPD. Results showed that early QFPD administration is linked to quicker recovery, decreased hospital stay, and decreased virus shedding time. In addition, the mortality rate was 0.3%, which is much lower than the global average mortality, proving the effectiveness of QFPD in treating COVID-19 (Shi et al., 2020). Another study (Xin et al., 2020) found that combining QFPD with Western medicine can improve COVID-19 symptoms and lung inflammation (Xin et al., 2020). Furthermore, studies suggest that combining QFPD with alpha-interferon, oseltamivir, phosphate, chloroquine, arbidol, and ribavirin to treat COVID-19 can reduce hospitalization stays and improve clinical symptoms and lung CT (Hu et al., 2020; Yang et al., 2020). Zhang et al. (2021) found that QFPD administration can decrease the mortality ratio. It is important to note that QFPD administration did not raise the risk of organ injury. A study of 76 COVID-19 patients from Hebei province, China, has reported that QFPD can control clinical symptoms and improve blood parameters with few adverse reactions (Rahaman et al., 2019).

XBFD is a novel TCM recipe formulated particularly for COVD-19 treatment with different TCM components, which have been proven effective against previously reported coronaviruses (BL, 2020). The clinical application of XBFD against COVID-19 has significantly decreased COVID-19 clinical symptoms such as cough, fever, fatigue, etc. and prevented the progression of mild/moderate disease to severe (Pan et al., 2020). In another report, 280 COVID-19 patients were treated with XBFD, and all cases recovered, preventing the transition from severe to critical cases (ChinaDaily, 2020). In addition, XBFD combined with conventional therapy can relieve symptoms of COVID-19, increase the patient’s WBC and lymphocyte count, and decrease erythrocyte sedimentation rate and C-reactive protein (Xiong et al., 2020).

A therapeutic process of 23 severe COVID-19 patients showed that treatment with HSBD can significantly reduce the probability of patients being admitted to the ICU and needing an artificial oxygen supply. Compared with Western medicine treatment, HSBD combined with the other three TCM injections (Xiyanping, Xuebijing, and Shenmai) has higher antiviral activity at different follow-up times and can also relieve inflammation in the lungs (Li et al., 2020; Wang et al., 2021a). Another mentioned that HSBD reduced viral load by 30% in mouse lung tissue and improved clinical symptoms (Li et al., 2020). In addition, combining HSBD with Western medicine (lopinavir-ritonavir) to treat COVID-19 can have better efficacy (Shi et al., 2021). It has been reported that HSBD has recovered severely infected COVID-19 patient symptoms, decreased disease severity, and minimized the mortality rate of critically infected patients (Pan et al., 2020).

JHQG has been widely used in COVID-19 patients (Ang et al., 2020; National Health Commission of the People’s Republic of China, 2020a; Huang et al., 2021). JHQG was administered to 80 COVID-19 patients and found that JHQG treatment reduced nucleic acid test negative conversion time. Furthermore, it can decrease inflammation in the lungs and increase the number of leukocytes and granulocytes significantly compared to control (Liu et al., 2020b). In another study, JHQG was used combined with Western medicine to treat 123 mild COVID-19 patients, and it was observed that JHQG combined with Western medicine could significantly improve COVID-19 symptoms such as fever, cough, fatigue, expectoration, etc., with gastrointestinal side effects such as diarrhea (Duan et al., 2020).

XBJ is a TCM injectable drug that has been extensively used for critical COVID-19 patients and has decreased inflammation storm and increased the blood oxygen level (Pan et al., 2020), which are two typical symptoms in critical COVID-19 cases (Huang et al., 2020b). XBJ was injected into 42 mild and severe COVID-19 cases for a week, and CT reports showed that 60% of the patient’s lung capacity had improved, the time of nucleic acid becoming negative was shorter, and thus the patient’s stay in the hospital had also been shortened. Moreover, the patient’s CBC reports showed that the patient’s white blood cell, neutrophil, platelet, and fibrinogen counts were increased. Meanwhile, the patient’s interleukin (IL)-6, IL-10, CRP, and d-dimer levels also decreased after treatment (Guo et al., 2020). Another study that compared the XBJ group (XBJ + routine medicine) with the control group (0.9% NaCl + routine medicine) showed that XBJ could cut down the rate of ARDS and artifactual supplication. Moreover, it can also reduce septic shock and stop disease progression from severe to critical. The cardinal symptoms (fever, cough, fatigue, and shortness of breath) in the XBJ-treated group were shorter than those in the control group, and they remained in the ICU for a lesser time compared to the control. Furthermore, IL-6, IL-8, and tumor necrosis factor alpha (TNF-α) counts were significantly reduced, without any anaphylactic shock (Luo et al., 2021). Zhang et al. (2020b) reported that lung inflammation could be absorbed better by treating it with XBJ. It has been explained in an *in vitro* study that XBJ use keeps cells safe from virus-induced cell death. Furthermore, it also protects the normal size of cells and inhibits plaque count in XBJ-treated cells in a dose-dependent manner compared to the control (Ma et al., 2020). Another study that used XBJ on 31,913 patients reported that the probability of side effects was 0.3% (Ma et al., 2020).

Recently, LHQW was administered to 245 COVID-19 patients. The results showed that it could effectively improve clinical symptoms (fever, cough, and fatigue). Furthermore, it also stops the disease’s progression (Zhuang et al., 2021). In addition, LHQW, compared with arbidol, can recover lung inflammation with no serious side effects (Yu et al., 2020). Compared with conventional therapy, LHQW treatment can improve fever more effectively (Lv et al., 2020; Yao et al., 2020; Zeng et al., 2020). One clinical retrospective analysis recruited 154 COVID-19 patients treated with LHQW and found that the probability of symptom remission was higher than the control group (Zeng et al., 2020). In another study, LHQW was administered to 142 COVID-19 patients for 14 days, and it revealed that LHQW has a significantly increased recovery rate and lung CT (Hu et al., 2021). LHQW was used on 51 moderate COVID-19 patients plus conventional therapy, and results showed that it has a high rate of fever improvement and a low rate for the illness getting worse; furthermore, LHQW also improved the lung CT images (Cheng et al., 2020). Xiao et al. (2020) reported that COVID-19 patients treated with the combination of LHQW, Huoxiang Zhengqi, and Western medicine could recover clinical symptoms by improving the efficacy of anti-infective drugs and prognosis.

## The Possible Mechanism of Action of the Three Chinese Medicines and Three Chinese Recipes

SARS-CoV-2 enters into the host through virus spikes and human ACE2 (hACE2) interaction called receptor-binding domain (RBD), proteolytically activated by cell surface protease TMPRSS2, lysosomal proteases cathepsin, etc. (Zhou et al., 2020; Sun et al., 2021). Furthermore, this entry pattern contributes to the rapid spread and onset of severe symptoms and high fatality of infected patients. Therefore, ACE2 and TMPRSS2 are considered candidates for the development of antiviral agents. To date, multiple (both TCMs and Western) drugs have shown a direct or indirect antiviral effect on COVID-19, but most of the available anti-COVID-19 drugs’, particularly TCMs, mechanism of action *in vitro* or *in vivo* is yet unclear. Multiple components of the three Chinese medicines and three Chinese recipes have downregulated the expression of ACE2 by regulating different transcription factors (Niu et al., 2020; Niu et al., 2021). Additionally, some reports have highlighted that different components of three Chinese medicines and three Chinese recipes have inhibited the replication of SARS- CoV-2 (Huang et al., 2021). Here, we summarize the three Chinese medicines and three Chinese recipes’ predicted mechanisms of action individually (Table 3).

**TABLE 3:** Possible mechanism of action of the National Health Commission (NHC)-approved Chinese drugs against COVID-19.

Drugs	Direct targets	Indirect targets	Pathways	References
QFPD	ACE2, 3CLpro, and PLpro	AKT1, MAPK1, MAPK14, IL-6, and TNF	NF-κB, TNF, PI3K-Akt, and MAPK	Chen et al. (2020), Xu Tian-fu and Yang (2020), Ren et al. (2021)
HSBD	ACE2, 3CLpro, and PLpro	IL-6, MAPK8, MAPK1, and IL-1β	TNF, PI3K-Akt, NOD-like receptor, MAPK, and HIF-1	Lai et al., 2020. (2020), Tao et al. (2020), Ren et al. (2021)
LHQW	ACE2, 3CLpro, and PLpro	IL-6, TNF, MAPK1, IL-1β, MAPK8, CCL2, CASP1,3, NLRP3, NFKB1, NF-κB, etc.	Toll-like receptor, NOD-like receptor, IL-17, TNF, and MAPK	Wenpan Peng et al. (2020), Ren et al. (2021), Yan and Zou (2021)
JHQG	ACE2, 3CLpro, and PLpro	IL-6, IL-1β, CXCL8, CCL2, IL-2, IL-4, ICAM1, IL-10, IFNG, IL-1A, etc.	PI3K-Akt, TNF, and Toll-like receptor	Qi and Wang (2016), Simayi MN et al. (2020), Ren et al. (2021)
XFBD	ACE2 and 3CLpro	IL-6, MAPK3, MAPK1, MAPK14, MAPK8, IL-1β, CCL2, EGFR, PPARG, NOS2, TTR, AKT1, FYN, TP53, rep, etc.	Toll-like receptor, TNF, NF-κB, MAPK, and IL-17	Wang et al. (2020), Wang et al. (2021b)
XBJ	ACE2 and 3CLpro	AKT1, IL-6, TNF, TP53, CASP3, IL-10, MAPK8, MAPK1, LI1B, CCL2, IL-4, etc.	Toll-like receptor, NF-κB, PI3K-Akt, and T-cell receptor	Dai et al. (2020), Ren et al. (2021), Tianyu and Liying (2021), Xing et al. (2020), Zheng et al. (2020)

QFPD decoction in English means lung cleansing and detoxifying decoction (Cao et al., 2020). ACE, ACE2, AGTR1, FURIN, TNF, CASP3, CASP6, DPP4, CL1, and POLD1 have also been predicted as QFPD targets in treating COVID-19 (Yan et al., 2020). Another report, based on the molecular docking technique, mentioned that QFPD could directly act on the 3CLpro and block its multiplication and ACE2 to reduce virus entry into cells (Wu et al., 2020). It has been suggested that QFPD has the highest number of targets for binding with various transcriptional factors (Chen et al., 2020). A total of 217 QFPD composition-related targets and 200 COVID-19 related targets were predicted. Finally, 51 related targets, including FOS, IL-6, CCL2, and IL-1α, were declared key targets (Xu et al., 2020). It has been reported that QFPD significantly reduced the release of IL-6 from macrophages by activating both LPS and poly (I: C)/Pam3CKS4. Therefore, it exerts moderate anti-inflammatory effects through the Toll-like receptor pathways (Yang et al., 2020). It has been further reported that the Toll-like signaling pathway has a vital role in the mechanism of QFPD action against COVID-19 treatment. Different QFPD components may directly interact with the Toll-like receptor four and control the mitogen-activated protein kinase (MAPK) signaling pathway and nuclear factor kappa B (NF-κB) signaling pathway to inhibit the secretion of IL-1β, TNF-α, IL-8, etc. (Yang et al., 2020). In COVID-19 patients, the QFPDD treatment demonstrated induced immunity, anti-inflammation, and organ protection, elucidating the QFPDD formula’s application in the SARS-CoV-2 campaign. According to one study, 12 bioactive components in QFPDD identified 623 targets, 88 of which overlapped with genes affected by SARS-CoV-2 infection. These target sites involve biological processes such as hemostasis; cell growth; pattern recognition receptor signaling; IL signaling; and COVID-19-related nervous, circulatory, and digestive system injuries. A pathway analysis also revealed 55 critical targets for QFPDD, which regulated five functional modules, including induced immunity, anti-inflammation, and organ protection. Baicalin, hesperidin, hyperoside, and glycyrrhizic acid were linked to seven key targets: AKT1, HMOX1, IL-6, IL-10, PTGS-2, TNFα, and TP53. Molecular docking analysis revealed that key components in QFPDD might bind to six host proteins, which then interact with COVID-19 virus proteins, thereby supporting QFPDD’s anti-SARS-CoV-2 effect (Zhao et al., 2021b).

Wang et al. (2020) predicted that there are 99 active compounds in XFBD, among which 22 can bind to ACE2 and 3CLpro proteins with binding properties similar to ribavirin, lopinavir, and ritonavir. Finally, IL-6, MAPK3, MAPK1, MAPK14, MAPK8, IL-1β, CCL2, EGFR, PPARG, and NOS2 were declared key targets of XFBD for COVID-19 (Wang et al., 2020). TTR, AKT1, FYN, and TP53 have been proposed as the main targets for XFBD; additionally, TP53 has a strong relationship with COVID-19 (Wang et al., 2021b). A clinical retrospective analysis of 42 COVID-19 patients suggests that XFBD together with conventional medicine can effectively relieve clinical symptoms, including cough, fever, shortness of breath, fatigue, etc. (Xiong et al., 2020). Furthermore, they also found that XFBD treatment also significantly raised lymphocytes and white blood cell count and decreased C-reactive protein and erythrocyte sedimentation rate (Xiong et al., 2020). Another report found that XFBD treatment has recovered severe COVID-19 cases with 13 days of treatment (Zhou et al., 2021b).

HSBD can be linked with ACE2 and 3CLpro (Lai et al., 2020; Xie et al., 2020). MAPK3, MAPK8, TNF, IL-6, and TP53 were declared the main targets of HSBDF for the treatment of COVID-19 (Tao et al., 2020). Other research initially predicted 45 potential targets for HSBD. Finally, RELA, TNF, IL-6, IL-8, MAPK14, TP53, CXCL8, MAPK3, MAPK1, IL-4, MAPK8, CASP8, and STAT1 were declared key targets of HSBD for the treatment of severe COVID-19 patients (Zhu et al., 2021). By comparing different studies, we found that IL-6, TNF, RELA, MAPK1, MAPK8, CXCL8, IL1B, MAPK14, TP53, CCL2, IL-2, PTGS2, and IFNG were the key targets for HSBD treatment of COVID-19 (Lai et al., 2020; Liu et al., 2020; Tao et al., 2020; Xie et al., 2020; Niu et al., 2021; Zhu et al., 2021).

Shen et al. (2020) suggested that HSP90AA1, AKT1, RELA, ACE2, and 3CLpro were also the five key targets for JHQG for the treatment of COVID-19. Using the DrugBank database, Lin et al. (2020) predicted 263 QFPD targets, compared them with 346 COVID-19 possible targets, and found 49 identical targets. Among them, IL-6, IL-1β, CXCL8, CCL2, IL-2, IL-4, ICAM1, IL-10, IFNG, and IL-1A are seen as important targets of JHQG for treating COVID-19 (Lin et al., 2020), and all of them are related to immune regulation (Chen et al., 2019). Peng et al. (2020) reported 720 potential targets for JHQG and predicted 253 disease targets by using the GeneCard and OMIM databases. Finally, they predicted 79 possible treatment targets; among them, 18 key targets were obtained, including MAPK1, MAPK3, TP53, JAK1, FOS, CASP3, IFNG, TNF, IL-1β, CXCL8, NFKB1, IL-6, PIK3CA, MAPK8, RELA, CASP8, IL-2, and MAPK14 (Peng et al., 2020). The TCMSP and SwissTargetPrediction databases found 414 treatment targets for QHQG, while the GeneCards database suggested 346 disease targets. Finally, 47 targets were shortlisted, among which eight were the core targets (MAPK1, CASP3, TP53, ALB, TNF, IL-6, MAPK8, and MAPK14) of QHQG for COVID-19 treatment (Mao et al., 2020).

It has been predicted that three XBJ ingredients (quercetin, beta-sitosterol, and luteolin) can combine with ACE2 and 3CLpro to achieve an antiviral effect (Dai et al., 2020). Another study suggested that XBJ can attach to 3CLpro, ACE2, and S protein (Xing et al., 2020). Zhao et al. identified 44 active ingredients, after comparing the potential targets of XBJ with COVID-19 targets, and they suggested 44 treatment targets such as AKT1, TP53, TNF, JUN, EGFR, IL-1β, IL-10, and EGF. Among them, AKT1 was identified as the core target, while two more active ingredients, luteolin and quercetin in XBJ, have a broad-spectrum effect on COVID-19 (Tianyu and Liying, 2021). Another study predicted 52 potential targets of XBJ for treating COVID-19, including IL-6, TNF, TP53, CASP3, IL-10, MAPK8, MAPK1, LI1B, CCL2, IL-4, etc., and it is thought to be involved in immune responses (Dai et al., 2020). According to another report, there were 144 potential therapeutic targets of XBJ against COVID-19, of which GAPDH, TNF, CASP3, EGFR, MAPK1, PTGS2, STAT3, and MAPK8 had high degree values by PPI network analysis. Moreover, these targets involved multiple inflammations and immune-related issues (Zheng et al., 2020).

LHQW contains a total of 13 TCM compounds; therefore, it is hard to describe the composite mechanisms of each component of LHQW action against COVID-19. A total of 160 active components were found in LHQW, with 57 target proteins, such as IL-6, MAPK1, HSP90AA1, CCL2, CASP1, NLRP3, NFKB1, and TNF, linking 35 signaling pathways such as the Toll-like receptor signaling pathway and the NOD-like receptor signaling pathway (Yan and Zou, 2021). Another study reported a total of 224 chemical components were obtained in LHQW, for 246 targets, such as IL-6, TNF-α, CASP3, NF-j B, and signaling pathways, MAPK, IL-17, and TNF (Wenpan Peng et al., 2020).

Two retrospective observational studies (Cheng et al., 2020b; Yao et al., 2020) found that combining LHQW with a conventional drug significantly accelerated the relief of fever, cough, and fatigue in COVID-19 patients. At the molecular level, LHQW inhibited SARS-CoV-2 replication and pathogenesis by reducing pro-inflammatory cytokine production (Runfeng et al., 2020). In clinical trials, LHQW administration is an effective treatment for COVID-19 patients. A combination of high-resolution mass spectrometry and an untargeted data mining approach analyzed LHQW components in treated COVID-19 patient plasma and urine. This combination identified 132 bioactive components in LHQW that humans absorbed *via* the gastrointestinal tract. The pharmacological characteristics of eight components identified in LHQW with a potential affinity for ACE2 receptors were further evaluated using data from comprehensive ACE2 screening using chromatography. The results show that forsythoside-A, forsythoside-I, rhein, neochlorogenic acid, and other components have a strong inhibitory effect on ACE2. This study investigated the molecular therapeutic mechanisms of LHQW treatment, demonstrating the utility of the COVID-19 patient exposure-based approach in identifying bioactive components in TCMs (Chen et al., 2021).

## Conclusion and Future Recommendations

The three Chinese medicines and three Chinese recipes are effective in the treatment and prevention of COVID-19 infections. In China, the three Chinese medicines and three Chinese recipes were used to treat COVID-19 infections, significantly alleviating disease symptoms, delaying mild disease progression to a severe stage, improving cure rates, and decreasing mortality rates. The three Chinese medicines and three Chinese recipes have been demonstrated to be highly effective against COVID-19, but there is a lack of evidence. Available literature analysis, *in silico*, and other types of studies suggest that the three Chinese medicines and three Chinese recipes may have antiviral, immune regulation, organ protection, and anti-inflammation actions *via* multi-component-based drugs that act on multiple targets in the host by using multiple pathways for COVID-19 treatments. However, most of the available data related to the potential mechanism of the three Chinese medicines and three Chinese recipes is based on virtual simulation, which is acquired *via* molecular docking and network pharmacology analysis, where different researchers employed different components, targets, and pathways to predict the possible mechanism of action (Table 3). These predictions have not yet been proven. Therefore, there is a need for high-quality *in vitro* and *in vivo* and clinical studies by employing new strategies and technologies, such as genomics, metabolomics, and proteomics, to verify the predicted mechanisms of these drug’s effects on COVID-19.

There has been a continuous debate for many decades among the world scientific community about the quality standards, pharmacodynamics, effective material basis, unclear toxicity, and efficacy of TCM. The ongoing COVID-19 pandemic and TCM therapeutic effects have once more become the bone of contention. Therefore, China’s national and provincial governments should initiate different projects to explore active components of TCMs, their mechanisms of action, quality control standards, TCM efficacy evaluations, and clinical studies with the help of modern industrial technologies.
